# Interferon alpha-inducible protein 27 (IFI27) is a prognostic marker for pancreatic cancer based on comprehensive bioinformatics analysis

**DOI:** 10.1080/21655979.2021.1985858

**Published:** 2021-10-21

**Authors:** Shu Huang, Jinglin Zhao, Jianxin Song, Yanqiong Li, Rongxia Zuo, Yalian Sa, Zhihui Ma, Hongmei OuYang

**Affiliations:** a The Affiliated Hospital of Kunming University of Science and Technology, Kunming, Yunnan, China; b Institute of Laboratory Medicine, The First People’s Hospital of Yunnan Province, Kunming, Yunnan, China; c Central Sterile Supply Department, The First People’s Hospital of Yunnan Province, Kunming, Yunnan, China; d Center for Clinical Medicine Research (Yunnan Provincial Key Laboratory of Clinical Virology), The First People’s Hospital of Yunnan Province, Kunming, Yunnan, China; e Institute of Ageing Research, School of Medicine, Hangzhou Normal University, Hangzhou, Zhejiang, China

**Keywords:** Interferon alpha-inducible protein 27 (IFI27), pancreatic cancer, tumor immune microenvironment (TIME), glycolysis, prognosis

## Abstract

Accurate biomarkers to predict the genesis and progression of pancreatic adenocarcinoma (PAAD) are needed in the fight against this deadly disease. Here, we combined multiple datasets (GEO, TCGA and GTEx) to conduct a comprehensive analysis of pancreatic cancer. Through an in-depth analysis, we discovered that the expression of the gene encoding interferon alpha-inducible protein 27 (IFI27) was significantly higher in pancreatic cancer tissues than that in normal tissues, and that higher expression of IFI27 was negatively correlated with the overall survival rate of pancreatic cancer patients. The functional annotation of IFI27 demonstrated relationships to cellular immunity and metabolism, especially glycolysis. Analysis of infiltrating immune cells displayed that higher expression of IFI27 expression correlates with decreased CD8 + T cells and increased M2 macrophages in the tumor immune microenvironment (TIME), then biochemical analyses of a mouse model and immunohistochemical (IHC) staining verified that glycolytic enzymes and M2 macrophages increased significantly in pancreatic cancer tissues. We speculate that IFI27 may affect the tumor microenvironment (TME) of PAAD by regulating cellular immunity and metabolism, thereby promoting the progression of pancreatic carcinoma and worsening the prognosis. These findings of our present study are solid evidence that IFI27 is a potential prognostic biomarker of pancreatic cancer and that it affects the tumor immune microenvironment.

## Introduction

Pancreatic adenocarcinoma (PAAD) is a kind of gastrointestinal tumor with high degree of malignancy, which is difficult to diagnose and treat, and has seriously threatened human life and health. The incidence rate of PAAD continues to increase yearly, with an estimated 60,430 new cases and 48,220 deaths worldwide in 2021[1]. Its prevalence is related to consumption of alcohol, smoking, high fat diets, excessive coffee consumption, environmental pollution and genetic factors[1][2]. It also often occurs in patients with diabetes or chronic pancreatitis [1,3].

PAAD is a relatively dangerous type of cancer. Its morbidity and mortality have exaggerated considerably over the past few years. The 5-year survival rate of pancreatic cancer patients is less than 10%, thus making pancreatic carcinoma one of the most malignant of the gastrointestinal tumors [3]. The onset of pancreatic cancer is quite subtle, and the malignant progression is quite rapid, meaning that once the cancer is discovered, in most cases, few effective treatment options remain. Therefore, the identification of new tumor biomarkers to diagnose pancreatic cancer accurately and rapidly would be useful in the battle against this deadly disease.

Recently, the mining of information available in public databases has yielded important insights and novel diagnostic biomarkers. With the deepening of tumor research, changes of tumor immune microenvironment have been found to play an essential role in the genesis, progression and prognosis of tumor. A recent study from a melanoma clinical trial suggested that anti-PD1/PD-L1 based therapy is more effective than traditional chemotherapy, suggesting that the tumor immune microenvironment (TIME) may have a certain regulatory effect on tumor [4]. Tumor infiltrating immune cells are one of the main components of the tumor microenvironment (TME). The TME, includes both immune cells and stromal cells [5], which are closely related to tumor cell proliferation, treatment response and prognosis.

Interferon alpha-inducible protein 27 (IFI27) is a hydrophobic mitochondrial protein composed of 122 amino acids [6]. Studies have reported that the gene encoding this protein, IFI27, is expressed at low levels in a variety of mammalian cells under normal conditions and that IFI27 participates in varieties of biological processes, including innate immunity and apoptosis [7,8]. In addition, multiple studies have shown that IFI27 is highly expressed in a number of cancers, such as ovarian cancer [9,10], hepatocellular carcinoma [11] and breast cancer [12]. In animal and cell models, the down-regulation of IFI27 can promote apoptosis induced by TNF-related apoptosis-inducing ligand in hepatocellular carcinoma and gastric cancer, suggesting that IFI27 may be a key protein for tumor development [13]. While IFI27 has been shown to be a key gene in several types of cancer, studies have not yet identified relationships between IFI27 expression and the progression of pancreatic carcinoma.

In this work, we utilized multiple datasets, including The Cancer Genome Atlas (TCGA), Gene Expression Omnibus (GEO) and Genotype-Tissue Expression (GTEx), and discovered that IFI27 is highly expressed in pancreatic cancer, and that pancreatic cancer patients with high IFI27 expression had worse survival rates. Gene Set Enrichment Analysis (GSEA) showed that IFI27 may be involved in glycolysis, myc targets v2 and mTORC1 signaling, thereby promoting progression of pancreatic cancer. In addition, we found that IFI27, as a new biomarker, may affect the tumor microenvironment, especially by promoting the decrease of CD8 + T cells and the increase of M2 macrophages. The content of this article will help us have a better understanding of the progression mechanism of pancreatic cancer, provide a new marker target for pancreatic cancer, and provide a new idea for the diagnosis and treatment of pancreatic cancer.

In brief, we aimed to identify potential immune-related biomarker in PAAD and attempted to elucidate the underlying mechanisms using bioinformatic approaches. IFI27, a biomarker significantly associated with survival status and clinical progression were identified. Meanwhile, combined with immune cell infiltration analysis and gene function enrichment analysis, we were capable of further demonstrate the new diagnostic marker for PAAD patients and provide new ideas for the clinical diagnosis and treatment of PAAD.

## Material and methods

### Gene expression data collection and processing

The microarray gene expression files we used were retrieved from the GEO database (www.ncbi.nlm.nih.gov/geo) using the subsequent four criteria:(a) Pancreatic cancer; (b) Human;(c) Series; and (d) Expression profiling by array. This search revealed 284 distinctive GEO series (GSEs). Microarray data GSE55643 (Submission date: 6 March 2014) was manually selected and obtained from the GPL6480 Platform. This dataset included 45 pancreatic ductal adenocarcinoma samples and 8 normal pancreatic tissues [14]. The microarray dataset GSE15471 [15,16] and GSE16515 [17–19] were also downloaded from the GPL570 Platform. GSE15471 (submission date 31 March 2009) consisted of 36 cases of pancreatic ductal adenocarcinoma tumors and 36 matched cases of normal pancreatic tissue samples, while GSE16515 (Submission date: 9 June 2009) included 36 pancreatic tumor and 16 normal samples. The TCGA-PAAD project, with 178 pancreatic adenocarcinoma patients and corresponding clinicopathological information, was acquired from the Genomic Data Commons Data Portal (https://portal.gdc.cancer.gov). This dataset, included both the ‘Counts’ files and the ‘Clinical’ files.

Considering the small number of normal pancreatic samples in TCGA datasets, the gene expression spectra for 167 normal human pancreatic tissues in GTEx were downloaded from the University of California, Santa Cruz (UCSC) Xena project (https://xenabrowser.net/datapages)(Table S1). For standardization, 0.01 was added to all gene expression values, and these values were then log2-transformed with the ‘limma’ package in R, version 3.6.1^21^.

### Screening of differentially expressed genes (DEGs) between tumor and normal tissues

The normalized expression profile of each GEO dataset was constructed via the ‘limma’ package and the ‘impute’ package in R (version 3.6.1) [20,21]. Overlapping up-regulated DEGs of GSE55643, GSE15471 and GSE16515 were selected by Venny 2.1.0, and matrices were constructed with logFoldChange (FC) >1 and adjusted *p*-value <0.05. We then combined the TCGA-PAAD and GTEx datasets to verify the 10 intersecting up-regulated genes, and the results were visualized with the ‘pheatmap’ package in R.

### Identification and validation of the differential expression hub gene

To obtain the hub differential expression genes (DEGs) in pancreatic carcinoma, we investigated the clinical information of the ‘TCGA-PAAD’ dataset and obtained a cohort containing 177 pancreatic cancer patients with their survival information. Then, analysis of overall survival (OS) was conducted with ‘survival’ package in R, the screening criterion was *p* < 0.05.

GEPIA2.0 (http://gepia2.cancer-pku.cn/) is an enhanced website server for analyzing the RNA-Seq expression data from TCGA and GTEx database, that includes a quantity of tumor and normal samples [22]. Kaplan‐Meier plots and correlation point plots were obtained from GEPIA2. Additionally, a box plot using the disease state as the abscissa was obtained to reckon the expression difference of IFI27.

Kaplan-Meier Plotter (http://kmplot.com/analysis) is a public database using Kaplan-Meier algorithm for prognosis analysis, which can be used to analyze the survival rate of more than 54,000 genes (including mRNA, miRNA and protein) in 21 kinds of tumors [23]. We utilized this tool to validate OS rate of patient samples in PAAD based on the expression of IFI27, *p* < 0.05 was considered statistically significant.

The Oncomine database (http://www.oncomine.org) is a tumor chip database that integrates RNA-seq and DNA-seq data from GEO, TCGA and published literatures and supports various analysis methods, including meta-analysis, interactome analysis and molecular concepts analysis [24]. Thus, we verified the expression of IFI27 in various tumors using Oncomine. In addition, we adopted one database to further validate the expression of IFI27 in the lacobuzio-Donahue Pancreas cohort of human pancreatic carcinoma.

### Pathway functional enrichment analysis of IFI27

We performed pathway enrichment analysis using normalized RNA-Seq data acquired from the TCGA-PAAD portal. We extracted the expression data of PAAD tumor samples and divided them into two sets according to the median expression of IFI27. The Gene Oncology (GO), Kyoto Encyclopedia of Genes and Genomes (KEGG) and hallmark gene sets functional enrichment analysis of Gene Set Enrichment Analysis (GSEA) were conducted by GSEA4.0.2 to examine possible biological functions of the gene products of IFI27 [25]. To enhance the statistical significance of the enrichment results, the number of permutations was set to 1,000, and the enrichment results had to meet two standards; a false discovery rate (FDR) less than 0.050 and a nominal *p*-value less than 0.050. ‘GSVA’ package (version 1.36.2) was applied to calculate the enrichment score of a specific gene set in each sample [26]. The single-sample GSEA (ssGSEA) scores were ultimately used for survival analyses of the functional pathways of each hallmark gene set.

### Immune cell infiltration analysis of IFI27

TIMER(https://cistrome.shinyapps.io/timer) is an extensive website resource for systematic review of infiltrated immune cells in different cancer types [27]. We assessed IFI27 expression in PAAD and its correlation with the abundance of immune cells in gene module, including B cells, CD4^+^ T cells, CD8^+^ T cells, neutrophils, macrophages and dendritic cells. A plot was generated via TIMER illustrating the correlation of IFI27 expression levels with tumor purity and immune infiltrated cells.

CIBERSORT (http://cibersort.stanford.edu) uses a deconvolution algorithm to analyze immune cell infiltration based on gene expression [28]. Using CIBERSORT, we measured 22 tumor infiltrated immune cells to evaluate their association with the expression of IFI27 in PAAD. The TCGA-PAAD ‘Counts’ files were used to obtain a gene expression matrix, and the default signature matrix was set at 1,000 permutations. To analyze the influence of IFI27 on the tumor immune microenvironment, 178 PAAD tumor samples were classified into high-IFI27 and low-IFI27 groups. To determine the levels of confidence in the identities of immune cells affected by IFI27, the CIBERSORT result was filtered with *p*-value <0.05. The survival and correlation analyses were conducted in R.

### Cell culture and construction of a mouse orthotopic model

The KPC cell line is an in vitro model of mouse pancreatic ductal adenocarcinoma cells, and it has multiple characteristics that are similar to those in human pancreatic cancer. KPC cells were cultured in RPMI 1640 medium containing 10% FBS and 1% penicillin-streptomycin at 37°C and 5% CO2. Cells were used within 10 generations. PCR was performed to verify that the cells were not contaminated by mycoplasma. 8-week-old female C57BL/6 mice were purchased from Charles River (Beijing, China). An in situ pancreatic tumor model was developed as previously described [29]. Briefly, subcutaneous xenograft tumors were generated by injecting 5×10^5^ KPC cells in 50 μL PBS subcutaneously into the dorsal flank of the mice. After incubation for 4 weeks, the mice were killed by dislocated method, and the tumor tissue was excised and made into 3×3*3 mm^3^ tumor lumps and placed in RPMI 1640 medium. Then, mice were anesthetized with 3% pentobarbital sodium to expose the abdominal cavity, and the tumor block was implanted into the tail of pancreas. The entire process was operated in a sterile environment. After three weeks, the tumor and the normal pancreas tissue were removed and stored in tissue preservation solution.

### Real-time quantitative PCR (qRT-PCR) analysis

The mRNA of markers of M2 macrophages and glycolysis hub gens were measured by qRT-PCR. Total RNA was separated using TRIzol reagent (Invitrogen, USA) according to the manufacturer’s instructions. The quality and concentration of RNA were determined using a Nano-100 micro-spectrophotometer (Allsheng, China). cDNA was reverse-transcribed from RNA using PrimeScript RT Master Mix (TaKaRa, Japan). For expression analysis, PCR was performed with TB Green™ Premix Ex Taq™ II (TaKaRa, Japan) in a Mx3000P Real-Time fluorescence quantitative PCR system (Agilent, USA), and 18SrRNA was used as an internal control. Changes were determined using the 2^−ΔΔct^ calculation method, and all measurements were performed in triplicate. See Table 1 for primer information.

**Table 1.** Primers information

Gene name	Primers sequences	Species
18SrRNA	F: 5ʹ-ATGCGGCGGCGTTATTCC-3’	Mouse
	F: 5ʹ-GCTATCAATCTGTCAATCCTGTCC-3’	
IFI27	F: 5ʹ-GACTCTCCGTGCCATCTACTG-3’	Mouse
	R: 5ʹ-CCTCTATCGCCATATCTGCCAC-3’	
CD163	F: 5ʹ- CTGGCGGGTGGTGAAAACA-3’	Mouse
	R: 5ʹ- CAGCCGTTACTGCACACTG-3’	
CD206	F: 5ʹ- GAGGGAAGCGAGAGATTATGGA-3’	Mouse
	R: 5ʹ- GCCTGATGCCAGGTTAAAGCA-3’	
HK2	F: 5ʹ- TGATCGCCTGCTTATTCACGG-3’	Mouse
	R: 5ʹ- AACCGCCTAGAAATCTCCAGA-3’	
LDHA	F: 5ʹ-TGTCTCCAGCAAAGACTACTGT-3’	Mouse
	R: 5ʹ-GACTGTACTTGACAATGTTGGGA-3’	
PGK1	F: 5ʹ-ATGTCGCTTTCCAACAAGCTG-3’	Mouse
	R: 5ʹ-GCTCCATTGTCCAAGCAGAAT-3’	
ENO1	F: 5ʹ-TGCGTCCACTGGCATCTAC-3’	Mouse
	R: 5ʹ-CAGAGCAGGCGCAATAGTTTTA-3’	

### Immunohistochemistry (IHC) staining

Human pancreatic tissue samples were collected from patients of RuiJin Hospital, Shanghai Jiaotong University, and the experimental protocols have been approved by the Ethics Committee of RuiJin Hospital, Shanghai Jiaotong University (Approval number: 2021–194). Tissues were fixed with 4% paraformaldehyde, then embedded and sliced in paraffin. The sections were dewaxed and heated in the Improved Citrate Antigen Retrieval Solution (Beyotime Biotechnology, P0083) for antigen repair and permeabilized with Immunostaining Permeabilization Buffer with Triton X-100(Beyotime Biotechnology, P0096). Endogenous peroxidase was blocked with Endogenous Peroxidase Blocking Buffer (Beyotime Biotechnology, P0100A). The sections were incubated with mouse anti-CD163 antibody (Cell Signaling Technology, 1:400) or anti-LDHA antibody (Cell Signaling Technology, 1:400). Antibody binding was detected with HRP-labeled secondary antibody (Santa Cruz Biotechnology Inc.) and visualized by the DAB+ Substrate Chromogen System (Dako Omnis). Samples were counterstained with hematoxylin, incubated in ethanol and xylene solution of ascending concentration, and finally fixed.

### Statistical analysis

All statistical analyses were performed in R 3.6.1 and GraphPad Prism 8 software. All statistical tests with *p* < 0.05 were considered to indicate statistical significance.

## Results

We conducted a comprehensive analysis of data from TCGA, GEO and GTEx, and we found that a significantly increased expression of IFI27 in tumor tissues was associated with worse overall survival in patients with PAAD. In addition, the ‘CIBERSORT’ algorithm and single-gene function enrichment analysis also showed that IFI27 is related to immune cell infiltration and glycolysis in the tumor microenvironment. A mouse orthotopic cancer model was utilized to demonstrate that IFI27, glycolysis and M2 macrophages were significantly up-regulated in pancreatic cancer. This study thus identified IFI27 as a potential prognostic marker and therapeutic target for PAAD.

### Identification of DEGs after data integration

**Figure 1.**
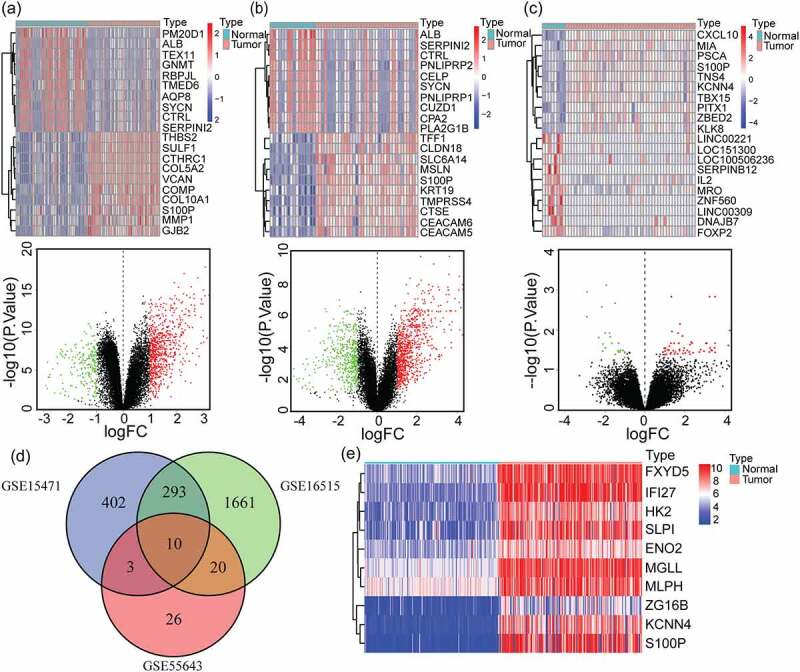
Identification of 10 overlapping up-regulated DEGs in PAAD. (a-c) Heatmaps of top 20 DEGs of pancreatic adenocarcinoma compared with normal tissues in GSE15471(a), GSE16515(b) and GSE55643(c) datasets (up), the red represents high expression and the blue represents low expression. Volcano plots of DEGs of pancreatic cancer compared with normal tissues in GSE15471(a), GSE16515(b) and GSE55643(c) datasets (down), the red represents high expression and the green represents low expression. GSE15471 identified 708 up-regulated genes and 210 downregulated genes. GSE16515 identified 1984 up-regulated genes and 3869 downregulated genes. GSE55643 identified 59 up-regulated genes and 23 downregulated genes. (d) Venn diagram of the 10 overlapping DEGs of GSE15471, GSE16515 and GSE55643. (e) Heatmap of validation of the expression of 10 overlapping DEGs combined with TCGA-GTEx pancreatic cancer datasets. DEGs, differentially expressed genes; PAAD, pancreatic adenocarcinoma; TCGA, The Cancer Genome Atlas; GTEx, Genotype-Tissue Expression Project

To investigate the key gene signature for pancreatic cancer, we performed a differential expression analysis via the R package ‘limma’. Heatmaps and volcano plots were created, in which red color represents genes with relatively high expression and blue and green colors represent genes of low expression (Figure 1(a-c)). Analyses of gene expression profiles from GSE15471 identified 918 DEGs, with 708 genes up-regulated and 210 genes down-regulated in PAAD tumor samples compared with normal control tissues. From the GSE16515 dataset, we recognized 5,843 DEGs, of which 1,984 genes were up-regulated and 3,859 genes were down-regulated in PAAD. A count of 82 DEGs were obtained from GSE55643. 59 genes were up-regulated and 23 genes were down-regulated in PAAD.

Grounded on the cutoff criteria, we obtained 10 overlapping up-regulated DEGs via the ‘VennDiagram’ package from the above mentioned three GEO datasets, including ENO2, KCNN4, ZG16B, MGLL, HK2, FXYD5, MLPH, SLPI, IFI27 and S100P (Figure 1(d)). Gene expression profiles of normal and tumor samples in the TCGA-GTEx cohort were utilized to construct a heatmap to confirm the overexpression of these 10 overlapping genes in PAAD samples (Figure 1(e)). The figure showed that compared with normal pancreatic tissues, IFI27 displayed up-regulation in tumor tissues.

### Identification and validation of key gene signature of IFI27 in PAAD

**Figure 2.**
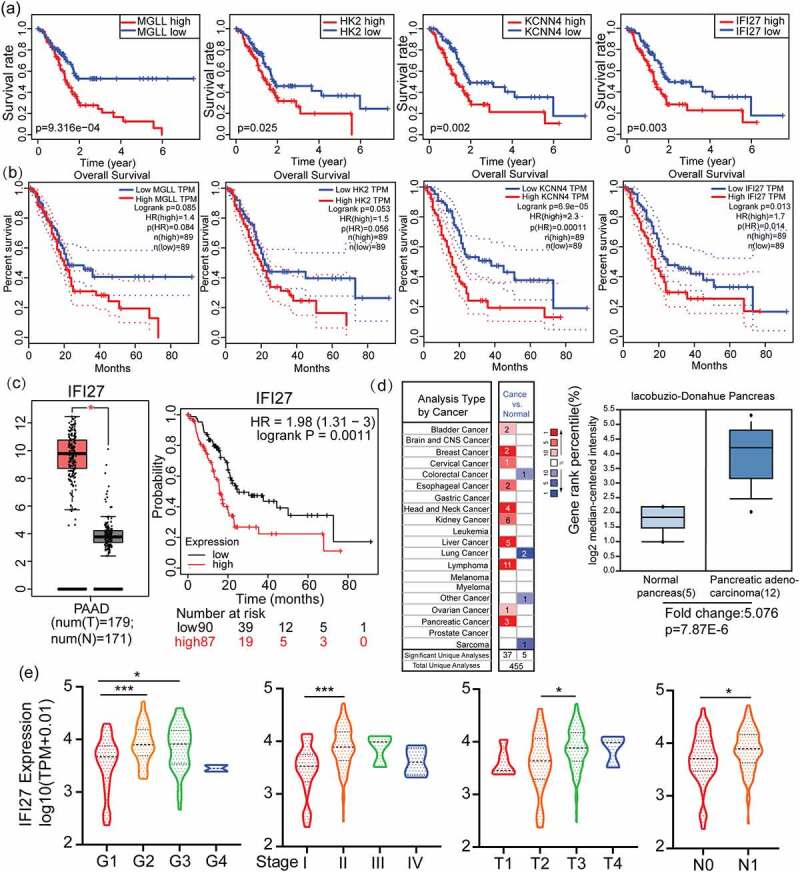
Identification and verification of IFI27 as a hub gene in PAAD. (a) The relationship between 10 overlapping DEGs expression levels and overall survival TCGA-PAAD. Only 4 genes (MGLL, HK2, KCNN4 and IFI27) with significant survival significance (*p* < 0.05) were displayed. (b) K-M plots of above 4 genes to verify the survival significance. (c) IFI27 was significantly up-regulated in PAAD compared with normal tissues. *p < 0.05 (left). The relationship between IFI27 expression levels and overall survival in PAAD analyzed by Kaplan-Meier Plotter (right). (d) mRNA levels of IFI27 were up-regulated in various kind of tumors compared with normal tissues (left). Box plot validating the expression of IFI27 in lacobuzio-Donahue Pancreas dataset based on the Oncomine database (right). (e) Correlations betweenIFI27 and clinical characteristics. *p < 0.05, **p < 0.01, ***p < 0.001. TCGA, The Cancer Genome Atlas; PAAD, pancreatic adenocarcinoma; DEGs, differentially expressed genes

In order to further explore the significance of these ten genes on the prognosis of pancreatic cancer, survival analysis was conducted to explore the significance of the ten genes on the prognosis of pancreatic cancer. The results showed that the expression of four genes had significant statistical differences on overall survival rate, including MGLL (*p* = 9.316 × 10–4), KCNN4 (*p* = 2.4 × 10–3), IFI27 (*p* = 3.309 × 10–3) and HK2 (*p* = 2.459 × 10–2) (Figure 2(a)).

GEPIA2.0 was utilized to further confirm the connections between OS and the expression of MAGG, KCNN4, IFI27 and HK2 in PAAD. As shown in Figure 2(b)), IFI27 (*p* = 0.014) and KCNN4 (*p* = 0.00011) are both factors that are significantly associated with poor prognosis. In other words, the high expression of IFI27 or KCNN4 is predictive of worse OS in PAAD. Notably, in investigating previous research exploring gene expression in PAAD, we found that IFI27 has only rarely been identified as a DEG of significance.

In addition, by using the GEPIA2.0 website tool, we confirmed that IFI27 is expressed higher in pancreatic cancer samples than that in adjacent normal tissues (*p*-value < 0.05, |Log2FC| > 1) (Figure 2(c)), left). Moreover, Kaplan-Meier Plotter was applied to verify the survival significance of the key gene IFI27 with *p* = 0.0011 (Figure 2(c)), right). Importantly, analysis using the Oncomine tool demonstrated that IFI27 is significantly increased in most types of cancer (Figure 2(d)), left). Compared with normal pancreas tissue samples, significantly upper level of IFI27 mRNA was observed in the cohort of pancreatic adenocarcinoma from Oncomine (Figure 2(d), right).

We used correlation analysis to evaluate the relationship between the expression of IFI27 and clinicopathological characteristics in PAAD patients. After excluding some samples without sufficient clinical information, we obtained 168 samples from TCGA that contain both IFI27 expression data and relevant clinical characteristics. As illustrated in Figure 2(e)), increased expression of IFI27 was significantly correlated with histologic grade (G2 vs. G1, *p* < 0.001; G3 vs. G1, *p* < 0.05), T stage (T3 vs. T2, *p* < 0.05), N stage (N1 vs. N0, *p* < 0.05) and tumor stage (stage II vs. stage I, *p* < 0.001). These results indicate that PAAD patients with high expression of IFI27 are more inclined to have pancreatic adenocarcinoma that are more advanced in histologic grade, T stage, N stage and tumor stage compared to those with low levels of IFI27. The above results showed that, consistent with the previous description, IFI27 is a biomarker of poor prognosis.

### Correlation of IFI27 expression with glycolysis

**Figure 3.**
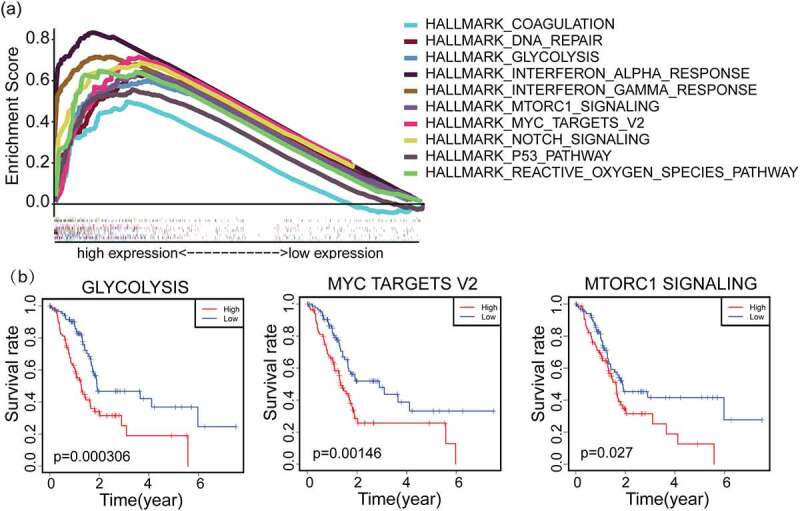
GSEA hallmark functional annotation of IFI27. (a) The top ten enriched pathways in PAAD samples with high expression of IFI27 were identified by GSEA. (b) Kaplan-Meier curves for glycolysis, myc targets v2 and mTORC1 signaling with higher IFI27 expression after filtered (*p* < 0.05). GSEA, Gene Set Enrichment Analysis; PAAD, pancreatic adenocarcinoma

GSEA was performed to investigate probable biological function of IFI27. GSEA uncovered significant differences (FDR < 0.050, *p*-value < 0.050) in functional enrichment pathways in samples with high expression of IFI27. We selected the most significantly enriched signaling pathways based on their corresponding normalized enrichment score (NES) ranking. As shown in Figure 3(a) and Table S2, hallmark enrichment analysis uncovered 10 positively correlated terms, including interferon alpha response, interferon gamma response, Notch signaling, mTORC1 signaling, glycolysis, myc targets v2 and so on. Then, these top ten pathways were further filtered by survival analysis according to their ssGSEA scores, and the three up-regulated pathways shown in Figure 3(b) (glycolysis, myc targets v2 and mTORC1 signaling) were found to statistically significant by survival analysis. These results indicate that the pathways regulating glycolysis, myc targets v2 and mTORC1 signaling, which may be vital in PAAD patients, were strongly associated with IFI27 expression.

GO and KEGG analyses also revealed the top ten functional enrichment pathways that were related to IFI27 (Figure S1). However, only a single enrichment pathway named ‘KEGG_GLYCOLYSIS_GLUCONEOGENESIS’ was found to related upon hallmark enrichment analysis. Taking previous studies reporting that cancer cells tend to rely on glucose metabolism into account, we focused on the pathway of glycolysis. Studies have shown that there are two main biochemical events in glucose metabolism of cancer cells: (i) increased glucose uptake and (ii) aerobic glycolysis, energy metabolism disorder or change has been recognized as one of the ‘hallmarks of cancer’[30]. Furthermore, our correlation analysis of IFI27 with key genes in glycolysis demonstrated that expression levels of genes coding for several key glycolytic enzymes, hexokinase 2 (HK2), phosphoglycerate kinase 1 (PGK1), alpha-enolase (ENO1) and lactate dehydrogenase A (LDHA), were significantly correlated with expression of IFI27 (Figure 5(a)).

### Correlation of M2 macrophages with the expression of IFI27

**Figure 4.**
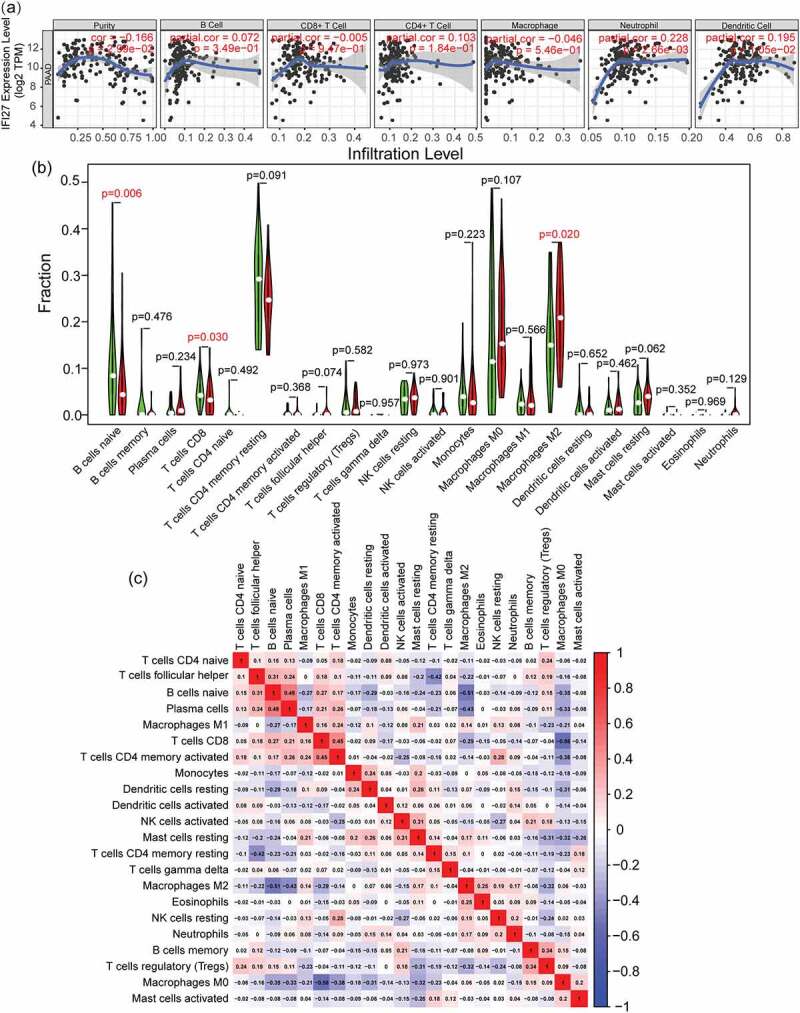
Tumor infiltrating immune cell analysis of IFI27. (a) Scatter plots showing the correlation of tumor infiltrating immune cells with IFI27 expression level generated by TIMER. (b) Wilcoxon rank sum test accurately compared the differences between the two groups, indicating that several immune cells in the high IFI27 group had significantly different infiltration density. (c) The relationship between the abundance ratios of ` 22 immune cells. The value represents the correlation value. Red represents a positive correlation, and the blue represents a negative correlation

Independent tumor-infiltrating immune cells play an important role in cancer progression and the prediction of overall survival rate. Therefore, TIMER was utilized to explore the potential relationship between IFI27 expression and the abundance of infiltrated immune cells in pancreatic cancer. As shown in Figure 4(a), the expression of IFI27 is positively correlated with the infiltration abundance of B cells (*p* = 2.99 × 10 − 2), Neutrophil (*p* = 2.66 × 10 − 3) and Dendritic cells (*p* = 1.05 × 10 − 2). These results imply that IFI27 may play a crucial role in tumor immune microenvironment in pancreatic carcinoma.

**Figure 5.**
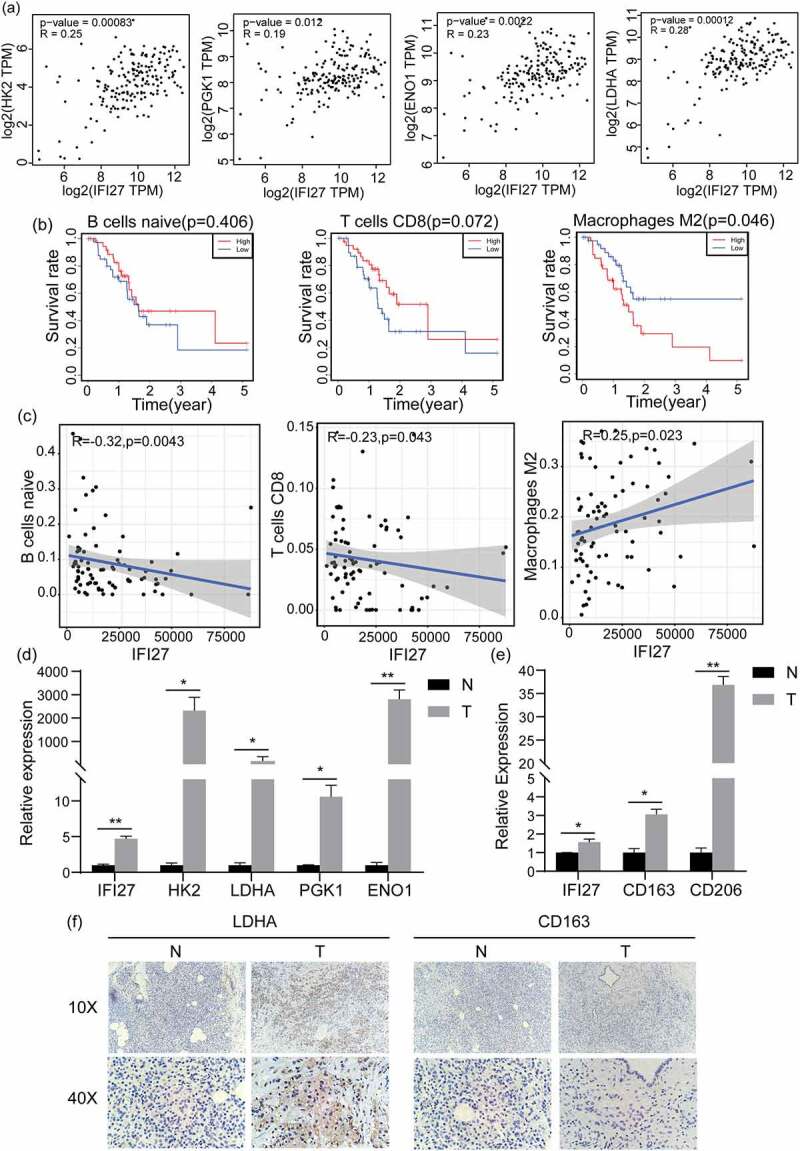
IFI27 is positively correlated with the expression of key genes in glycolysis and M2 macrophage markers. (a) Pearson’s correlation analysis of IFI27 and glycolysis-related genes in GEPIA2.0. (b) Survival analysis of infiltrated immune cells. (c) Scatterplots representing the relationships between the expression of IFI27 and infiltrated immune cells in TCGA. (d) mRNA expression levels of glycolysis-related genes in C57BL/6 mice pancreatic cancer and normal pancreatic tissues measured by qRT-PCR. (e) mRNA expression levels of M2 macrophage markers in pancreatic cancer and normal pancreatic tissues of C57BL/6 mice. (f) Immunohistochemical staining for LDHA (left) and CD163 (right) was performed in pancreatic cancer. *p < 0.05, **p < 0.01, ***p < 0.001. N, normal pancreatic tissues; T, pancreatic tumors; GEPIA, Gene Expression Profiling Interactive Analysis; TCGA, The Cancer Genome Atlas; qRT-PCR, quantitative real-time PCR

In addition, we wished to gain a measure of how different the TIME of patients with high IFI27 expression in pancreatic cancer is entirely different from that of patients with low expression of IFI27. As compared to the median expression of IFI27, 178 pancreatic tumor specimens were divided into two groups: a high-expression group (89 cases) and a low-expression group (89 cases). Then, the CIBERSORT deconvolution algorithm was applied to the 22 immune cell subtypes, which aided in evaluating the difference of immune infiltration between high and low IFI27 expression groups. Naive B cells, CD8 + T cells and M2 macrophages were significantly influenced by IFI27 expression. Compared with the group with low IFI27 expression, M2 macrophages (*p* = 0.02) were distinctively increased in the high IFI27 group, while CD8 + T cells (*p* = 0.03) were obviously decreased (Figure 4(b)). The possible correlations with 22 types of immune cells were also evaluated. The resulting heatmap reclosed a weak to moderate correlation between the 22 immune cells in the invasion of pancreatic adenocarcinoma (Figure 4(c)). In addition, the 5-year survival analysis also indicated that M2 macrophages (*p* = 0.046) were negatively correlated with the survival of pancreatic cancer (Figure 5(b)). IFI27 expression also displayed a significantly negative correlation with naive B cells (R = −0.32, *p* = 0.004) and CD8 + T cells (R = −0.32, *p* = 0.004), while IFI27 showed a positive correlation with M2 macrophages (R = 0.25, *p* = 0.023; Figure 5(c)).

### Up-regulation of IFI27 in pancreatic cancer

We compared the mRNA expression of IFI27 in pancreatic cancer tissues from a KPC-generated orthotopic mouse model to the expression in normal mouse pancreatic tissues (Figure 5(d,e)). The results obviously suggest that IFI27 mRNA is expressed at a higher level in pancreatic tumor tissues. It’s increasingly evident that glycolysis increases the rate of glucose hydrolysis, and tumors are more inclined to this way of metabolism in tumors. Notably, Figure 5(a)) and Figure 5(c)) showed that the expression of IFI27 is significantly and positively correlated with glycolysis pathway, which is known to be central to tumor metabolism [31], and with M2 macrophages, which are involved in the growth and progression in various types of cancer [32]. Therefore, qRT-PCR was used to determine the key glycolysis-related key genes (Figure 5(d))) and M2 biomarkers (Figure 5(e))) were both up-regulated in pancreatic carcinoma compared with normal tissues. In addition, we detected the expression of LDHA and CD163 in pancreatic cancer tissues and paired para-tumor normal tissues by IHC staining (Figure 5(f)). The results showed that LDHA was significantly up-regulated in pancreatic cancer tissues compared with the controls, but. increased expression of CD163 in pancreatic cancer was not detected. In this case, further exploration is needed to clarify the relationships of the expression of IFI27 with glycolysis and M2 macrophages.

## Discussion

Pancreatic cancer is a highly malignant gastrointestinal tumor, and the survival is the lowest for the cancer of pancreas (10%) [33]. Therefore, finding a reliable prognostic marker for pancreatic cancer is an important pursuit. In our study, RNA-seq data from GEO and TCGA-GTEx were retrieved, along with the corresponding clinical data, to perform a comprehensive analysis of PAAD.

IFI27 (also known as ISG12 or p27) is located on human chromosome 14q32 and belongs to a family of small, interferon alpha-inducible genes. The specific function of IFI27 is still unclear [12]. The complimentary DNA for IFI27 was initially cloned as an estrogen-inducible gene in the human epithelial cell line MCF-7^34^. In situ hybridization to some of the IFI27-overexpressing tumors showed that IFI27 mRNA is localized in cancer cells and sometimes also in fibroblast cells of the tumor stroma [34]. However, the impact of IFI27 in the progression of pancreatic cancer remains to be elucidated.

It has been reported that the key gene that we uncovered, IFI27, is an oncogene that correlates with poor survival in cholangiocarcinoma. However, we have not found relevant studies reporting the effect of IFI27 on the prognosis of pancreatic cancer. It can be deduced from Figures 1 and 2 that compared with normal tissues, the expression of IFI27 is significantly higher in pancreatic cancer tissues, and the prognosis of patients with high expression of IFI27 is worse, which shows that IFI27 is a poor prognostic factor for pancreatic cancer. The GSEA enrichment analysis of IFI27 also shows that IFI27 is mainly involved in cellular immune responses and cell metabolism, which suggest that it may promote tumor progression.

There are also reports revealing that overexpression of IFI27 can induce epithelial–mesenchymal transition and promote the tumorigenicity, migration and invasion, stemness and drug resistance of epithelial ovarian cancer cells [10]. Accordingly, in our report, the clinical correlation analysis showed that the expression of IFI27 in pancreatic cancer with low malignancy was significantly lower than that of high malignant pancreatic cancer. Immune cell analysis showed that IFI27 has a significant effect on the immune cell infiltration of pancreatic cancer, especially CD8^+^ T cells, which are also named cytotoxic T-lymphocytes (CTL) and are reported to eliminate transformed tumor cells and regulates immune response [35], and the degree of CD8^+^ T cells infiltration is lower in the high IFI27 group. M2 macrophages have been reported to promote tumor progression [36], and we found that M2 macrophage infiltration is increased in patients with high IFI27 expression. At the same time, CD8^+^ T cells are more highly expressed in low-malignant pancreatic cancer samples, while M2 macrophages are highly expressed in high-malignant samples, which is consistent with the expression of IFI27 and also confirms that IFI27 correlates with pancreatic cancer progression and can serve as a poor prognostic biomarker, providing a novel potential strategy for the early diagnosis and prognosis of pancreatic cancer. Through our comprehensive analysis, we found that IFI27 is significantly increased in pancreatic cancer tissues, and pathway enrichment found that IFI27 is mainly related to cellular immunity and metabolism. It has been reported that the unique way that tumor cells digest glucose is called ‘Warburg’ metabolism, which means their metabolism also depends on glycolysis even if under aerobic conditions [37]. In the case of the enhanced metabolism of tumor cells, it may cause metabolic disorders of CD8^+^ T cells and reduce their functions. This result is also consistent with the decrease in CD8^+^ T cell infiltration in the high IFI27 group. Overall, in this study we found a new biomarker, IFI27, to assist the early diagnosis and prognosis of pancreatic cancer.

On the basis of abovementioned findings, we used public databases to study the biological function of IFI27 within pancreatic cancer. Our results revealed that upregulation of IFI27 in PAAD can promote the progression of pancreatic cancer. Moreover, we also found that the expression of IFI27 has a significant impact on the tumor immune microenvironment of pancreatic cancer. Nevertheless, in this article, despite our utilization of public database of pancreatic cancer data to briefly explore the possible tumor progression aspects of IFI27, including glucose metabolism and immune microenvironment, the exact molecular mechanisms linking IFI27 to PAAD and its role *in vivo* remains to be further studied. Additional explorations into the area might illuminate a brand-new pathway that promotes pancreatic cancer progression.

## Conclusion

We identified a novel immune-related biomarker related to survival status, IFI27, using bioinformatic approaches and experimental validations. IFI27 was found to be closely related to the clinical progression of PAAD, as well as tumor glycolysis and infiltration of M2 macrophages.

Verification with a mouse orthotopic model verification showed that IFI27, glycolysis and M2 macrophages were significantly up-regulated in tumor tissues. Further exploration of relationships of IFI27 with glycolysis and M2 macrophages may provide new targets for the clinical diagnosis and treatment of PAAD.

## Supplementary Material

Supplemental Material

## Highlights

(1) IFI27 was identified as a biomarker related to immune cell infiltration and cell metabolism in PAAD.

(2) Differences of the infiltrated immune cell landscapes in PAAD were demonstrated.

(3) A therapeutic molecular marker IFI27 of PAAD was identified.
